# Linking Leader Humor to Employee Innovative Behavior: The Roles of Work Engagement and Supervisor’s Organizational Embodiment

**DOI:** 10.3389/fpsyg.2020.592999

**Published:** 2020-12-14

**Authors:** Jingjing Zhang, Weilin Su

**Affiliations:** ^1^Health and Social Research Center, Management Institute, Xinxiang Medical University, Xinxiang, China; ^2^School of Literature, Capital Normal University, Beijing, China

**Keywords:** leader humor, employee innovative behavior, work engagement, supervisor’s organizational embodiment, Chinese employee

## Abstract

The influence of leader humor on employee innovative behavior has been attracting increasingly more attention from various scholars and enterprises. Based on previous relevant literatures in the fields of humor, leadership, and innovation, this study proposes and verifies a model to examine the internal mechanism and boundary condition of the relationship between leader humor, work engagement, supervisor’s organizational embodiment, and employee innovative behavior. Specifically, this study introduces work engagement as a mediator in the relationship between leader humor and employee innovative behavior, and supervisor’s organizational embodiment as a moderator in the relationship between leader humor and work engagement. Then, this study conducts two separate questionnaire surveys on Chinese employees and their direct supervisors at two different times to collect the sample data. In total, 383 supervisor–subordinate dyads were collected. The results suggest that leader humor can promote employee innovative behavior. Work engagement can partly mediate the influence of leader humor on employee innovative behavior. Supervisor’s organizational embodiment of employee can positively moderate the influence of leader humor on work engagement, which in turn ultimately should account for positive increases of employee innovative behavior. The conclusions from the analyses above not only further verify and develop some previous points on leader humor and employee innovation but also derive certain management implications for promoting employee innovative behavior from the perspective of leader humor.

## Introduction

Humor, a social mode designed to make people feel funny, is ubiquitous in human beings’ work and daily life (Cooper, 2005; Mesmer-Magnus et al., 2012). Given its capacity to generate a series of positive outcomes in the workplace (Karakowsky et al., 2020), humor has been considered as a valuable tool for leadership purposes. As agents of the organizations, leaders usually maintain important power and core resources, so it is particularly important for leaders to show humor at work (Cooper et al., 2018; Kong et al., 2019). In fact, as a communication strategy to amuse employees (Pundt and Herrmann, 2015), leader humor has been proved to have a positive influence on employee’s emotion, attitude, performance, and other positive behaviors (e.g., Cooper, 2005; Hughes and Avey, 2013; Yam et al., 2018; Guenzi et al., 2019). Such influence has also been verified in the context of Western culture. For example, Pundt (2015) confirmed that German employees who perceived more humor from their leaders would showcase more innovative behavior. On the other hand, China has its own unique traditional culture, which deeply influences the way of thinking of Chinese employees and their expectations of leader humor (Yang et al., 2017). As a result, under the context of Chinese culture, whether how and when leader humor affects employee innovative behaviors is quite different from the cases in the western context. However, the existing researches have not provided any answer to this issue, so further exploration and verification are needed.

In addition, many previous studies have found that stimulating innovation and innovative behavior of employees is one successful way for enterprises to acquire and maintain their competitive advantages. This is also a reason why many companies strive to encourage their employees to innovate actively. Generally, employee innovative behavior refers to the activities related to innovation that employees actively participate in, including the generation, promotion, implementation, and retaliation of their innovative ideas (Janssen, 2003; Su et al., 2019). Shalley and Gilson (2004) have pointed out that innovative behavior of employee is characterized by high risks and high investment, which requires employees to be fully engaged and maintain a highly focused state of mind (Pieterse et al., 2010; Park et al., 2014). Prior research has also confirmed that a high level of work engagement means enough extra energy, enthusiasm, and continuous focus on work (Aryee et al., 2012), forming a crucial positive factor contributing to the tendency of individuals to take a variety of initiatives, including innovative behavior (Sonnentag, 2003; Eva et al., 2019). Besides, many previous scholars have demonstrated that when employees perceive humor from their leaders, they would be more deeply engaged in their work (Kim et al., 2016; Goswami et al., 2016). Similarly, this study also attempts to introduce work engagement as a mediator to explore the internal mechanism for leader humor to promote employee innovative behavior.

Furthermore, one of the most important implicit assumptions about the influence of leaders on employees is that the former can represent the organization (Hou et al., 2018; Su et al., 2019). Supervisor’s organizational embodiment (SOE), as a concept that describes employees’ perceptions about supervisors’ shared characteristics with the organization (Eisenberger et al., 2010), can enhance or weaken the influence of leaders on their employees’ attitudes and behaviors (Shoss et al., 2013; Mackey et al., 2018). Although supervisor’s organizational embodiment is relatively an under-researched construct in literatures on leadership and organizational behavior (Hussain and Shahzad, 2018, 2019), it is possible that this variable can moderate the process for leader humor to influence employees’ attitudes and behaviors. Hence, this study attempts to explore supervisor’s organizational embodiment as a moderator in the relationships between leader humor and employee work engagement, because this study believes that supervisor’s organizational embodiment of employees can positively moderate the influence of leader humor on their work engagement. To be specific, for employees with high supervisor’s organizational embodiment, leader humor would have greater impact on their work engagement. In contrast, employees with low supervisor’s organizational embodiment are less likely to be affected by the humor of their leaders and less likely to actively improve their work engagement.

**FIGURE 1:**
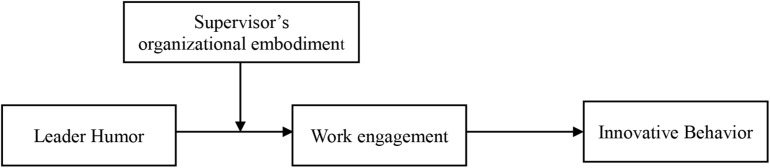
Theoretical model.

In summary, based on relevant existing literatures in the fields of humor, leadership, and innovation, this study attempts to explore the influence of leader humor on employee innovative behavior. Moreover, a model is built to examine the mediating role of work engagement in the relationship between leader humor and employee innovative behavior, as well as the moderating role of supervisor’s organizational embodiment in the relationship between leader humor and employee work engagement. The overall theoretical model of this study is shown in Figure 1.

## Theoretical Background

### Leader Humor and Employee Innovative Behavior

Leader humor, whether in the form of telling jokes or engaging in friendly banters (Karakowsky et al., 2020), refers to a kind of social communication behavior delivered by leaders to amuse employees and to be perceived by employees (Cooper et al., 2018), including playful verbal or non-verbal actions (Pundt and Venz, 2017). In a sense, leader humor is meant to amuse their subordinates (Cooper, 2008) and foster a positive communication atmosphere between leaders and employees (Kuiper et al., 2010). Most of the existing studies on leader humor have demonstrated that it is positively related to a range of positive work-related attitudes and behaviors of employees, such as work engagement (Yam et al., 2018), positive emotion (Robert and Wilbanks, 2012; Cooper et al., 2018), organizational commitment (Wells, 2008), and organizational citizenship behavior (Goswami et al., 2016). Therefore, following previous studies, this study infers that leader humor can effectively stimulate employee innovative behavior for the two reasons below:

First, scholars have proved that the process of making humor can reflect the creative thinking mode of leaders, and their humor is also regarded as a signal to encourage subordinates to break the routines (Holmes, 2007). From the perspective of employees, as recipients of leader humor, they can not only appreciate the humor of leaders (Yam et al., 2018) but also learn from the positive and optimistic attitudes of leaders (Cooper et al., 2018). Through these observations and learning, employees will be more confident in their ability to solve problems creatively (Su et al., 2019), while they will be more flexible in the face of difficulties and more likely to generate innovative ideas (Baas et al., 2013). In this sense, leader humor would spark more innovative behaviors among employees.

Second, leaders can use humor to share interesting things with employees, so as to create a relaxed and pleasant working atmosphere in their organizations (Kim et al., 2016), thus relieving employees’ perception of the risks and uncertainties related to the implementation of innovative behaviors (Ho et al., 2011; Pundt, 2015). Meanwhile, leader humor can facilitate employees to feel the trust and support from leaders, thus forming a high-quality relationship between supervisors and subordinates (Robert and Wilbanks, 2012; Cooper et al., 2018). In order to maintain this friendly relationship with leaders, employees will work harder to solve problems encountered at work, actively create new ideas, and try to enhance their work processes in innovative ways. Hence, this study formulates the following hypothesis:

Hypothesis 1: Leader humor is positively related to employee innovative behavior.

### Work Engagement as a Mediator

Work engagement refers to the degree of employees’ commitment, satisfaction, and enthusiasm for their jobs (Kahn, 1990; May et al., 2004), with an emphasis that employees would become more attached to their organizations in a long-term state. Originating from trust (Kahn, 1990), it has become a much broader concept over the past few decades (Luthans and Peterson, 2002), with many previous studies (e.g., Babcock-Roberson and Strickland, 2010; Tims et al., 2011; Su et al., 2019) confirming that leaders’ behavior or styles can affect their employees’ work engagement. Goswami et al. (2016) confirm that positive humor from leaders can promote their employees’ work engagement. In addition, when leaders leverage humor to share interesting things with their employees, the latter can personally feel the boost and support from their leaders (Kim et al., 2016), and in the spirit of reciprocity (Wu et al., 2006), these employees are bound to generate the willingness to reward their leaders and the organization they represent, thereby maintaining a higher level of work engagement.

As a dynamic and complex process, innovation entails a lot of trials and errors and continuous improvements based on knowledge, ability, and motivation (Amabile, 1997). Therefore, it requires individuals to commit enough time and abundant energy (April et al., 2019). Schaufeli et al. (2006) point out that individuals’ positive emotional experience, which is closely related to work engagement, can effectively expand their habitual thinking and activities, and widen their cognitive range, finally making their behaviors more flexible and creative. Salas-Vallina et al. (2020) also verify such positive emotions, finding that harmonious work passions of employees can lead them to engage in novel solutions and result in innovative work behavior. In a word, if employees dare to face various problems at work and devote themselves to searching for solutions, their creativity will be fully stimulated (Pieterse et al., 2010). The focus and persistence in this process will bring out more ideas and breakthroughs (Montani et al., 2020), while constantly motivating individuals to practice, thus exhibiting a high level of innovative behavior. That is to say, employees with high work engagement can fully mobilize the resources around them to meet the challenge encountered in their jobs with enthusiasm. As a result, they are more likely to generate innovative ideas at work and then try to find ways to realize such new ideas, thus driving more innovations.

Based on the analysis above, this study believes that leader humor can stimulate employee innovative behavior, because such humor can deepen their work engagement and deliver them more confidence and abilities to implement innovation. Therefore, this study proposes that work engagement would be a mediator in the relationship between leader humor and employee innovative behavior, as follows:

Hypothesis 2: Work engagement mediates the relationship between leader humor and employee innovative behavior.

### Supervisor’s Organizational Embodiment as Moderator

The concept of supervisor’s organizational embodiment was proposed by Eisenberger et al. (2010) to describe “the degree of employees’ perception of their leaders or supervisors as the organizational agent.” Employees often take supervisor’s organizational embodiment to measure their relationship with their organization (Eisenberger et al., 2014). In other words, employees with a high level of supervisor’s organizational embodiment usually attributes the care and encouragement of the leader to the willingness of the organization (Mackey et al., 2018), which means that they are more likely to interpret the exchange relationship between themselves and their supervisors as positive (Su et al., 2019). In this context, employees’ psychological needs, such as self-esteem, emotional support, and subjective well-being, would be easily satisfied (Eisenberger et al., 2010). As a result, employees are more willing to enhance their work engagement or even showcase more organizational citizenship behaviors, such as helping behavior, knowledge sharing behavior, and innovative behavior (Su et al., 2019).

As the research work go deep, many scholars begin to pay attention to how supervisor’s organizational embodiment amplifies the impact of leaders on employees, that is, the moderating role of supervisor’s organizational embodiment. For example, Shoss et al. (2013) indicate that supervisor’s organizational embodiment could moderate the relationship between abusive supervision and perceived organizational support. Hou et al. (2018) pinpoint that supervisors’ organizational embodiment can further amplify the influence of supervisors’ and organizations’ support on organizational citizenship behavior of employees. Su et al. (2019) verify that supervisor’s organizational embodiment can moderate the relationship between supervisor developmental feedback and employees’ creative self-efficacy. Following the similar logic here, this study suggests that the positive influence of leader humor on employee work engagement will be strengthened by supervisor’s organizational embodiment. Specifically, as an interpersonal communication strategy enhances interactions, leader humor would reflect their willingness to be active, please subordinates, and shorten the distance between supervisors and subordinates (Cooper, 2008). Enjoying leader humor, the employees with high supervisor’s organizational embodiment are more likely to interpret the humor as the will and behavior of the organization, so they tend to be affected and then make a positive response, such as a higher degree of work engagement. On the contrary, when levels of supervisors’ organizational embodiment are low, employees would believe that leader humor is only an expression of their personal characters, rather than representing the organization. Besides, this type of employees would not attach too much importance to the humor of supervisors, which therefore cannot effectively promote their work engagement. Hence, this study proposes the moderating role of supervisor’s organizational embodiment in the relationship between leader humor and work engagement to be as follows:

Hypothesis 3: Supervisor’s organizational embodiment moderates the relationship between leader humor and employee work engagement, such that this relationship would become stronger as supervisor’s organizational embodiment is obvious than when it is low.

## Materials and Methods

### Samples and Procedures

The sample data for this study were collected from employees and their immediate supervisors of five Chinese high-tech companies in three cities. These five companies are comparable in terms of several basic organizational characteristics: They are all IT-oriented organizations at a similar size and structure, located in eastern China. This study first contacted their human resource directors to get the names and E-mail addresses of the employees and their direct supervisors to be surveyed. The E-mail addresses of the participants were also used to pair the data. Then, adopting the suggestions of Podsakoff et al.’s (2003; see also Podsakoff et al., 2012), this study reduced the Common Method Variance and got the data at two different times from two-source survey with employee–supervisor matched. At time 1, the employees were asked to fill in the questionnaire via E-mail to measure their perceptions of leader humor (independent variable), work engagement (mediator), supervisor’s organizational embodiment (moderator), and their demographic information (e.g., age, gender, education, and work tenure with their direct supervisor). A total of 435 valid responses were obtained, representing a response rate of 87%. At time 2, 1 month later, via E-mail, this study invited the immediate supervisors of those employees surveyed at the first time to evaluate their subordinates’ innovative behavior (dependent variable) via E-mail. In the second round, at a response rate of 92.4%, 101 supervisors returned their completed surveys, rating 402 employees.

After cleaning up and matching the responses from the employees and their direct supervisors based on their E-mail addresses, this study finally got a sample of 383 employees, including 42.30% of males and 57.7% of females. In terms of age, most of them (89.4%) were below 40 years old and only 0.5% were 55 years old or higher. In terms of education, 56.7% had a bachelor degree, 27.4% a college degree or below, and another 15.9% a graduate degree or above. In addition, 51.7% of participants reported working with their leaders for less than 3 years, and 80.2% for less than 8 years.

### Measures

The original scales adopted by this study were all written in English, so they had to be translated to Chinese. This study adopted Brislin’s (1983) recommendation to back-translate these four scales. At first, two management scholars fluent in both Chinese and English were invited to translate all the items from English to Chinese. Then, another bilingual professor was invited to translate the Chinese version back into an English version. In order to improve the accuracy of the translation and avoid cultural bias, this study also implemented a small-scale pretesting to check the translation for the equivalence in meanings of all items. The final complete scales in this study are summarized below (Table 1).

**TABLE 1:** All the measures of studied variables.

Constructs	Items	Sources
Leader humor	My direct leader often uses humor in different situations when interacting with me.	Cooper et al. (2018)
	My direct leader often jokes around with me.	
	In general, my direct leader often expresses humor with me at work.	
Supervisor’s organizational embodiment	When my direct leader praises me, I feel like the organization praises me.	Eisenberger et al. (2010)
	When my direct leader is pleased with my work, I feel that the organization is pleased.	
	When my direct leader compliments me, it is the same as the organization complimenting me.	
	When my direct leader pays attention to my efforts, I believe that the organization is paying attention to my efforts.	
	My direct leader is characteristic of the organization.	
	My direct leader and the organization have a lot in common.	
	When I am evaluated by my direct leader, it is the same as being evaluated by the organization.	
	My direct leader is representative of the organization.	
	My direct leader is typical of the organization.	
Work engagement	I feel bursting with energy at work.	Schaufeli et al. (2006)
	I feel strong and vigorous at work.	
	When I get up in the morning, I want to go to work.	
	I am enthusiastic about my work.	
	My work inspires me.	
	I am proud of my work.	
	When I work hard, I feel happy.	
	I am immersed in my work.	
	I get carried away when I am working.	
Innovative behavior	This employee can come up with creative idea at work.	Scott and Bruce (1994)
	This employee can search out new technologies, processes, techniques, and/or product ideas.	
	This employee can promote and champion his/her ideas to others. This employee can investigate and secure funds needed to implement his/her new ideas.	
	This employee can develop adequate plans and schedules for the implementation of his/her new ideas.	
	In general, this employee is innovative.	

### Leader Humor

Employee’s perception of leader humor was measured with a 3-item scale developed by Cooper (2008). This scale had been verified in the Chinese context (Li et al., 2019). At Time 1, all employees were asked to rate statements based on their actual perception of humor from their direct supervisors. For example, one item stated “My direct leader usually uses humor in different situations when interacting with me.” A five-point Likert scale format (1 = strongly disagree, and 5 = strongly agree) was applied. The Cronbach’s α of this scale was found to be 0.741 in this study.

### Supervisor’s Organizational Embodiment

Supervisor’s organizational embodiment of employee was measured with a 9-item scale developed by Eisenberger et al. (2010). This scale had also been verified under the China’s cultural context by Hou et al. (2018) and Su et al. (2019). At Time 1, this study invited employees to assess how they feel about their direct supervisors acting as organizational agents. For example, one item stated “When my direct leader praises me, I feel like the organization praises me.” Again, a five-point Likert scale format (1 = strongly disagree, and 5 = strongly agree) was applied. The Cronbach’s α of this scale was found to be 0.912 in this study.

### Work Engagement

Work engagement of employee was measured by a 9-item scale developed and validated by Schaufeli et al. (2006) in ten different countries. This scale is a simplified cross-cultural version and had been verified with the Chinese samples by Fong and Ng (2012). For example, one item stated “When I work hard, I feel happy.” At Time 2 in this survey, all the employees were asked to rate the statements of work engagement on a five-point Likert scale format (1 = strongly disagree, and 5 = strongly agree). The Cronbach’s α of this scale was found to be 0.885 in this study.

### Employee Innovative Behavior

Employee innovative behavior was measured by a 6-item scale developed by Scott and Bruce (1994). At Time 2, this study invited supervisors to rate the innovative behavior of their direct employees. This scale had also been verified with Chinese samples (e.g., Hon, 2011; Si and Wei, 2012; Riaz et al., 2018). For example, one item stated “This employee can come up with creative idea at work.” All supervisors were invited to rate their subordinates on a five-point Likert scale format (1 = never, and 5 = always). The Cronbach’s α of this scale was found to be 0.914 in this study.

### Control Variables

Consistent with previous studies (e.g., Cooper, 2005; Pundt, 2015; Goswami et al., 2016; Hussain and Shahzad, 2019), this study selected gender, age, education, and tenure (which reflects work with the employment duration with the direct leader) of employees as the main control variables for their probable relationship with leader humor, work engagement, supervisor’s organizational embodiment, and innovative behavior of employees. The four control variables in this study were all collected from employees at Time 1.

## Results

### Confirmatory Factor Analysis

Before testing the three hypotheses proposed above, confirmatory factor analyses were conducted to examine whether supervisors’ evaluations of their subordinates’ innovative behavior and subordinates’ scores on their self-report scales (i.e., leaders’ humor, work engagement, and supervisors’ organizational embodiment) had captured the entire conceptual model. The results are presented in Table 2, which indicates that the 4-factor model (Model4: χ^2^/df = 2.023, RMSEA = 0.076, CFI = 0.944, TLI = 0.938, SRMR = 0.0652) fits the final data well. It is also better than other three alternative measurement models. Hence, this study concludes that the measures of four core variables in this study captured the distinct constructs.

**TABLE 2:** Results of CFAs: comparison of measurement models.

Models	Factors	χ^2^/*df*	RMSEA	CFI	TLI	SRMR
Model 1	One factor: LH + WE + SOE + EIB	7.651	0.132	0.630	0.594	0.178
Model 2	Two factors: LH + WE + SOE, EIB	4.344	0.094	0.796	0.814	0.119
Model 3	Three factors: LH, WE + SOE, EIB	3.941	0.083	0.838	0.820	0.088
Model 4	Four factors: LH, WE, SOE, EIB	2.023	0.076	0.944	0.938	0.052

**N* = *383. LH represents leader humor; WE represents work engagement; SOE represents supervisor’s organizational embodiment; EIB represents employee innovative behavior. Deal model-fit indicators are:*χ^2^*/df* < *3, RMSEA* < *0.08, CFI* > *0.9, TLI* > *0.9, SRMR* < *0.08.**

### Descriptive Statistics

The means, standard deviations, and bivariate correlations among control variables, i.e., leader humor, work engagement, supervisor’s organizational embodiment, and employee innovative behavior, are displayed in Table 3. As evidenced in Table 3, leader humor was positively related to employee innovative behavior (*r* = 0.30, *p* < 0.01) and work engagement (*r* = 0.41, *p* < 0.01). Meanwhile, work engagement is also positively correlated with employee innovative behavior (*r* = 0.31, *p* < 0.01). Taken together, these results provided preliminary support for the hypothesized relationships.

**TABLE 3:** Means, standard deviations, and bivariate correlations among studied variables.

Variables	Mean	SD	1	2	3	4	5	6	7
1. Gender	1.58	0.25							
2. Age	2.48	0.62	−0.14*						
3. Education	2.82	0.68	–0.03	0.13*					
4. Tenure	2.74	2.17	–0.09	0.36**	0.18*				
5. Leader humor	2.53	0.83	–0.04	–0.05	0.04	0.11*			
6. Work engagement	2.51	0.73	0.03	–0.02	–0.03	0.14*	0.41**		
7. Supervisor’s organizational embodiment	2.61	0.78	–0.05	–0.03	–0.02	0.09	0.50**	0.51**	
8. Innovative behavior	3.18	1.09	0.05	–0.06	0.19**	0.08	0.30**	0.31**	0.39**

*N* = 383; *p < 0.05, and **p < *0.01.*

### Hypothesis Testing

In order to test the hypotheses, this study implemented a hierarchical regression analysis with SPSS 22.0, as displayed in Table 4. After control over the employees’ gender, age, education, and work tenure, Model 5 indicates that leader humor is positively related to employee innovative behavior (β = 0.288, *p* < 0.001), so Hypothesis 1 is supported.

**TABLE 4:** Hierarchical regressions for main study variables.

Variables	Work engagement			Employee innovative behavior		
	Model 1	Model 2	Model 3	Model 4	Model 5	Model 6
Gender	0.034	0.052	0.096*	0.047	0.060	0.048
Age	–0.077	–0.031	–0.005	−0.109*	–0.077	–0.070
Education	–0.048	–0.058	–0.048	−0.189***	0.182***	0.195***
Tenure	0.177**	0.121*	0.071	0.092	0.052	0.024
LH		0.402***	0.167**		0.288***	0.195***
WE						0.233***
SOE			0.311***			
LH * SOE			0.279***			
*R*^2^	0.028	0.186	0.368	0.052	0.133	0.177
Δ*R*^2^		0.158	0.182		0.081	0.044
*F*	2.768*	17.238***	31.161***	5.399***	11.055***	13.477***

*N* = 383; LH represents leader humor; WE represents work engagement; SOE represents supervisor’s organizational embodiment; ***p < 0.001, **p < 0.01, *p < *0.05.*

For the mediating effect of work engagement, this study followed the procedures proposed by Preacher and Hayes (2008) to test the indirect influence of leader humor on employee innovative behavior via work engagement. As shown by Models 4, 5, and 6, after control over the effects of gender, age, education, and work tenure, leader humor was a significant direct predictor of employee innovative behavior (Model 5: β = 0.288, *p* < 0.001). After added work engagement to the hierarchical regression analysis model, work engagement could also significantly predict employee innovative behavior (Model 6: β = 0.233, *p* < 0.001); meanwhile, the influence of leader humor on employee innovative behavior is still significant (Model 6: β = 0.195, *p* < 0.001), suggesting that work engagement could partly mediate the influence of leader humor on employee innovative behavior. In order to analyze the indirect effect that leader humor has on employee innovative behavior through work engagement, this study used Bootstrap methods in virtue of PROCESS macros developed by Preacher et al. (2007) with Model 4. The results showed that the indirect influence of leader humor on employee innovative behavior through work engagement is significant (indirect effect = 0.108, with a 95% CI of [0.0605, 0.1725]). Hypothesis 2 is therefore well supported.

**FIGURE 2:**
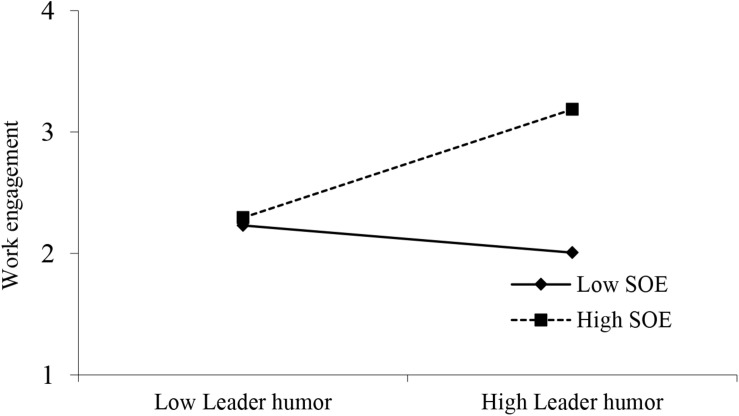
Interaction of leader humor and SOE on work engagement.

For the moderating effect of supervisor’s organizational embodiment in the relationship between leader humor and work engagement, this study adopted Hayes’s (2015) procedures for testing a moderating effect. After control over employees’ gender, age, education, and work tenure, as Models 1, 2, and 3, leader humor also becomes a significant predictor of employee work engagement (Model 2: β = 0.402, *p* < 0.001). Meanwhile, the interaction term of leader humor and supervisor’s organizational embodiment is significant in predicting work engagement (Model 3: β = 0.279, *p* < 0.001). Further, this study adopted the recommendations of Cohen et al.’s (2003) to plot this interaction as a conditional value of supervisor’s organizational embodiment (one standard deviation above and below the mean), as displayed in Figure 2. The results confirm that the direct influence of leader humor on work engagement is significant for employees with high supervisor’s organizational embodiment (*b* = 0.3415, 95% CI = [0.2360, 0.4470]; 1 SD above the mean), but not for employees with low supervisor’s organizational embodiment (*b* = −0.0291, 95% CI = [−0.1502, 0.0920]; 1 SD below the mean). Hence, Hypothesis 3 is well supported.

## Discussion

Leader humor is a hot topic in the field of leadership research (Cooper et al., 2018; Li et al., 2019), and its influence on employee innovative behavior has been attracting wide attention from various scholars and enterprises (Pundt, 2015; Goswami et al., 2016; Karakowsky et al., 2020). After reviews of literatures on humor and innovation, this study offers theoretical and empirical accounts for whether and how leader humor affects employee innovative behavior by assuming work engagement as a mediator and supervisor’s organizational embodiment as a moderator. Using multi-time data from pairing samples of 383 Chinese employees and their immediate supervisors, this study reveals that leader humor is positively associated with employee innovative behavior, and this relationship is partly mediated by work engagement. In addition, as expected, supervisor’s organizational embodiment acts as a moderator in the relationship between leader humor and work engagement to enhance the influence of leader humor on work engagement of employees.

### Theoretical Implications

The findings of this study contribute to the literatures on humor and innovation in several ways. First, this study suggests that leaders have a positive effect on employee innovative behavior. As mentioned above, the research on leader humor is still in its infancy. Although some prior studies have attempted to explore the relationship between leader humor and individual outcome variables (e.g., Cooper, 2008; Robert and Wilbanks, 2012; Kim et al., 2016; Yam et al., 2018), the understanding of leader humor in academic circles is still very limited (Pundt and Venz, 2017). In particular, the existing researches on leader humor are mainly conducted in the West context (Ho et al., 2011; Pundt, 2015), while the relevant studies in the Chinese context are still basically in blank (Li et al., 2019). Based on the samples of Chinese employees and their direct supervisors, this study reveals that leader humor can promote employee innovative behavior. As investigated so far, this study is the first to combine the leadership theory and the humor theory to explore the influence of leader humor on employee innovative behavior in the Chinese context, so it would enrich the existing literatures on leadership and innovation (Kim et al., 2016; Su et al., 2019; Hoang et al., 2020).

Second, this study reveals an influencing mechanism that transmits the effect of leader humor onto employee innovative behavior. As indicated by many scholars, the existing researches on leader humor are fragmented, mainly due to the lack of integrated theoretical framework (Yam et al., 2018; Guenzi et al., 2019). Therefore, it is very important to explore the influencing mechanism of leader humor from different theoretical perspectives (Goswami et al., 2016; Cooper et al., 2018). Inspired by this idea, this study introduces work engagement as a mediator variable, finding that humor leader can significantly affect work engagement, which in turn plays a part of the mediator role in the relationship between leader humor and employee innovative behavior. This finding would not only expand the research on the internal mechanism of leader humor and its incentive effect on employee innovation but also enrich the research on the positive effect of work engagement and its relationship with individuals’ innovative behavior. To some extent, this also responds to the call of Mao et al. (2017) and opens the “black box” in the process of leader humor motivating employee innovative behavior.

Third, this study explores an important boundary condition for the relationship between leader humor and work engagement. Our results suggest that supervisors’ organizational embodiment can positively moderate the relationship between leader humor and work engagement. In other words, for employees with a high level of supervisor’s organizational embodiment, leader humor may generate more forces to promote employees’ work engagement. Consistent with previous researches (Cooper, 2008; Robert and Wilbanks, 2012; Kim et al., 2016), this study further confirms that individual differences of employees can affect the degree to which leader humor would affect employees. Besides, to the best of our knowledge, this study is the first to introduce supervisors’ organizational embodiment as a moderator into the exploration of the relationship between leader humor and employee work engagement and verifies its applicability in the Chinese context. These results also respond to the appeals by many pervious scholars (e.g., Eisenberger et al., 2010; Stinglhamber et al., 2015; Su et al., 2019) to explore the role of supervisor’s organizational embodiment in the fields of Organizational Behavior and Management Psychology.

### Managerial Implications

Our results can also provide relevant and productive guidance for management. Firstly, by exploring the influence of leader humor on employee innovative behavior, this study provides practical reference value for companies and enterprises to leverage leader humor better. The increasing spiritual demands mean that employees will no longer be satisfied with the boring way of working (Yang et al., 2019; Chen and Li, 2020). To a certain extent, leaders can establish an interesting and free culture by offering humor in their organizations (Cooper et al., 2018), but the full play of the role of leader humor cannot be achieved without the support of their organizations. Hence, managers should pay more attention to the power of humor in management practice, while using humor as an effective management tool. For example, well-meaning jokes can be used to stimulate the potentials of employees. Besides, for leaders who are not humorous themselves, organizations can provide humor and humorous behavior related courses in leadership training, so as to further make them more humorous.

Secondly, the mediating role of work engagement indicates that employee innovative behavior is not only affected by leader humor but also affected by their own work engagement. Therefore, organizations need to realize the important role of employees’ psychological state and improve their work engagement. For example, managers can comprehensively boost employees’ positive psychology and behavior through humor, so as to encourage them to actively engage in work and finally facilitate their innovative behavior. Moreover, managers should deepen their understanding of employee work engagement, track and analyze the development trend of such engagement, and then take appropriate measures, such as job rotation and job redesign, to promote employee work engagement. In addition, this study suggests that organizations should create an inclusive and open internal environment by encouraging leaders to make more use of friendly jokes and other humorous ways to liven up the atmosphere when communicating with employees. These initiatives can help both leaders and employees to establish a good working relationship, stimulate employee work engagement, and motivate employees to exert their creativity in a free and relaxed work environment.

Thirdly, the moderating effect of supervisor’s organizational embodiment reflects that it is possible to further amplify the effect of leader humor on employee work engagement. Therefore, managers should improve supervisor’s organizational embodiment of employees by implementing diverse management practices that can advance the positive influence of leader humor on employees work engagement. Concretely, managers should constantly improve their professional abilities and management skills, earnestly understand and conscientiously implement the relevant rules and regulations of the organization, and pay attention to their words and deeds, so that they can become the real organizational agents in the eyes of employees. Meanwhile, organizations should clarify the legitimacy of leader’s identity, solidify the internal consistency between managers and organizations, and truly deliver the mutual integration between all staffers.

### Limitations and Future Research

Despite the implications above, this study has some limitations inevitably, some of which may inspire future research. First, the research method of this study fails to build a causal relationship among leader humor, work engagement, and employee innovative behavior. Although this study has collected the simple data from two sources at two different times to control the common method bias (Podsakoff et al., 2003, 2012), the measurements of leader humor, supervisor organizational embodiment, and work engagement were still measured by using participants’ self-perception. Moreover, the impact of leader humor on employees is a long-term dynamic process (Cooper, 2008; Kim et al., 2016; Li et al., 2019), so questionnaires cannot strictly test the causal relationship between related variables. Therefore, this study encourages future researches to adopt an experimental design to draw clear conclusions about causality.

Second, this study constructed and verified a model to examine the internal mechanism of the relationship between leader humor and employee innovative behavior, as well as the boundary condition of the relationship between leader humor and employee work engagement. On the one hand, this study only introduced work engagement as a mediator in the relationship between leader humor and employee innovative behavior. Nevertheless, this management phenomenon may be explained by other mediating mechanisms (Mesmer-Magnus et al., 2012; Lin, 2016), which can be explored by future studies from different theoretical perspectives, so as to deepen the understanding of this management problem. On the other hand, supervisor’s organizational embodiment was introduced only as one of many boundary conditions in the relationship between leader humor and work engagement. However, many previous studies have indicated that other variables, such as mindfulness (Montani et al., 2020), personal need for structure (Pundt and Venz, 2017), work meaning (Cai et al., 2018), and work autonomy (Li et al., 2019), could moderate the relationship between leader humor and employees’ actions. Hence, future researches could go further by incorporating other moderating variables to examine the boundary conditions of the relationship between leader humor and employee work engagement.

Finally, this study collected samples exclusively from Chinese companies, which may limit the generalization of our conclusions to specific cultural profiles. Previous studies have confirmed that cultural background may be an important factor affecting employees’ perception of leader humor (Yam et al., 2018) and supervisor’s organizational embodiment (Su et al., 2019). Specifically, compared with Western employees, Chinese employees are easily influenced by leader humor and are more likely to perceive leader as the embodiment of the organization. Therefore, future researches are encouraged to duplicate this study in other specific cultures, especially in the Western context.

## Conclusion

The present study has built and confirmed a conceptual model to uniquely combine leader humor and employee innovative behavior. Our conclusions suggest that leader humor positively affects employee innovative behavior via work engagement. Besides, supervisor’s organizational embodiment of employee moderates the direct influence of leader humor on work engagement, which, in turn, ultimately should account for positive increases of employee innovative behavior. Specifically, the above direct relationship between leader humor and work engagement is stronger in the employees with a higher level of supervisor’s organizational embodiment.

## Data Availability Statement

The raw data supporting the conclusions of this article will be made available by the authors, without undue reservation.

## Ethics Statement

The studies involving human participants were reviewed and approved by Ethical committee of Xinxiang Medical School, China. The participants provided written informed consent to participate in this study.

## Author Contributions

JZ and WS were responsible for and participated in the present study. JZ as the first author, designed the research and wrote and revised the manuscript. WS made some contributions in research design, data collection, data analysis, and critical revision. Both authors contributed to the article and approved the submitted version.

## Conflict of Interest

The authors declare that the research was conducted in the absence of any commercial or financial relationships that could be construed as a potential conflict of interest.
